# Meta-analysis of Inter-species Liver Co-expression Networks Elucidates Traits Associated with Common Human Diseases

**DOI:** 10.1371/journal.pcbi.1000616

**Published:** 2009-12-18

**Authors:** Kai Wang, Manikandan Narayanan, Hua Zhong, Martin Tompa, Eric E. Schadt, Jun Zhu

**Affiliations:** 1 Department of Genetics, Rosetta Inpharmatics, Seattle, Washington, United States of America; 2 Department of Computer Sciences, University of Washington, Seattle, Washington, United States of America; Duke University, United States of America

## Abstract

Co-expression networks are routinely used to study human diseases like obesity and diabetes. Systematic comparison of these networks between species has the potential to elucidate common mechanisms that are conserved between human and rodent species, as well as those that are species-specific characterizing evolutionary plasticity. We developed a semi-parametric meta-analysis approach for combining gene-gene co-expression relationships across expression profile datasets from multiple species. The simulation results showed that the semi-parametric method is robust against noise. When applied to human, mouse, and rat liver co-expression networks, our method out-performed existing methods in identifying gene pairs with coherent biological functions. We identified a network conserved across species that highlighted cell-cell signaling, cell-adhesion and sterol biosynthesis as main biological processes represented in genome-wide association study candidate gene sets for blood lipid levels. We further developed a heterogeneity statistic to test for network differences among multiple datasets, and demonstrated that genes with species-specific interactions tend to be under positive selection throughout evolution. Finally, we identified a human-specific sub-network regulated by *RXRG*, which has been validated to play a different role in hyperlipidemia and Type 2 diabetes between human and mouse. Taken together, our approach represents a novel step forward in integrating gene co-expression networks from multiple large scale datasets to leverage not only common information but also differences that are dataset-specific.

## Author Summary

Two important aspects of drug development are drug target identification and biomarker discovery for early disease detection, disease progression, drug efficacy and drug toxicity, etc. Recently, many single nucleotide polymorphisms (SNPs) associated with human diseases are discovered through large genome-wide association studies (GWAS). However, it is still largely unclear how these candidate SNPs may cause human diseases. The ultimate aim of this paper is to put these GWAS candidate SNPs and their associated genes into a network context to understand their mechanism of action in human diseases. In addition to large-scale human data sets that are often heterogeneous in terms of genetic and environmental factors, many high quality data sets in rodents exist and are frequently used to model human diseases. To leverage such information, we developed a method for combining and contrasting gene networks between human and rodents, specifically to elucidate how GWAS candidate SNPs may contribute to human diseases. By identifying mechanisms that are conserved or divergent between human and rodents, we can also predict which disease causal genes can be studied using rodent models and which ones may not.

## Introduction

The advent of expression profiling and other high throughput technologies has enabled us to systematically study complex human diseases by simultaneously measuring tens of thousands of molecular species in any given cell-based system [1]. It is now routine to organize such large-scale gene expression data into co-expression networks to shed light on the functional relationships among genes, and between genes and disease traits [2],[3],[4],[5]. Analysis of co-expression networks can be used to study any tissue or organ (such as liver, which plays a key role in the metabolism of glucose, lipids and toxic compounds), as long as the samples from such organs are collected in a population setting. Given that mouse and rat populations are commonly used to study human diseases in this manner, it is important to understand the mechanisms that are conserved between human and the rodent species, especially as we seek better predictions of the efficacy of drug targets identified from mouse or rat in human populations. In addition, identifying mechanisms that differ between humans and rodents can help to improve the design and interpretation of toxicity studies that involve rodent models.

Meta-analysis is the statistical synthesis of data by aggregating results from a set of comparable studies [6]. It can be used to systematically examine similarities and differences between molecular profiling studies carried out in populations from different species [7]. In a gene co-expression network, relationship between gene pairs is usually measured by correlation coefficients of different forms, such as Pearson correlation, Spearman correlation, or Mutual Information. Therefore, the problem of combining or comparing co-expression relationships across multiple datasets can be framed in the context of a meta-analysis of correlation coefficients, for which various methods have already been introduced. One method is Fisher's Inverse 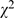 test, which computes a combined statistic (*S*) from the *p*-values of the correlation coefficients obtained from (*k*) individual datasets as, 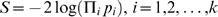. Under fairly general conditions this statistic follows a 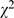 distribution with 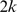 degrees of freedom under the joint null hypothesis of no correlation, making it possible to compute *p*-values of the combined statistic.

Another widely used meta-analysis method involves computing a weighted average of a common metric (i.e. effect size) derived from correlation coefficients in the individual datasets. Such statistic can then be used to test for homogeneity over the individual measures and for statistical significance. Datasets in this type of meta-analysis are typically weighted by the accuracy of the effect size they provide, which is a function of the individual sample sizes. Once the mean effect size is calculated, its statistical significance can be assessed by estimating the pooled variance of the mean effect size. In defining the effect size, Hedges and Olkin [8] and Rosenthal and Rubin [9] both advocated converting the correlation coefficient into a standard normal metric using Fisher's Z-transformation and then calculating a weighted average of these transformed scores. Depending on whether the effect sizes are assumed to be equal or not in the multiple datasets, fixed effect as well as random effect models can be employed. In the fixed effect models, the effect size in the population is a fixed but unknown constant and therefore is assumed to be the same for all datasets included in the meta-analysis. For random effect models, effect sizes may vary from dataset to dataset, and are assumed to be a random sample of all population effect sizes. Hunter and Schmidt [10] introduced a single random-effects method based on untransformed correlation coefficients. One important feature of this type of method is that heterogeneity of the effect sizes can be estimated, which provides a way to assess the difference in correlation coefficients across multiple datasets. Schulze [11] provided a thorough review of these meta-analysis methods and their applications.

For a meta-analysis of co-expression networks from diverse datasets, such as those constructed from different species, one central issue is that it is often unreasonable to assume that every gene pair has a unique, true effect size across evolutionarily diverse species. Although random effect models provide a more realistic way to accommodate cross species variation, it still assumes a parametric distribution on the population effect sizes. To circumvent this problem, a non-parametric meta-analysis method was introduced for the identification of conserved co-expression modules from human, fly, worm and yeast [7]. In this method, Pearson correlation coefficients of expression profiles between every gene pair were computed in each organism and then rank-transformed according to their correlations with all other genes. A probabilistic test based on order statistics was then applied to evaluate the probability of observing a particular configuration of ranks across the different organisms by chance. The advantage of this method is two-fold: 1) because the method is based on non-parametric statistics, it makes no assumption on the underlying distribution of correlation coefficients across multiple datasets; and 2) the effect size (i.e. the rank ratio statistic for every gene pair) is defined in a gene-centric fashion such that for any given gene, correlations with all other genes are considered. However, the method also has several limitations including 1) the loss of power in general given the non-parametric formulization [12],[13], and 2) the meta-analysis results cannot be represented in the same format as the individual datasets given there is no concept of a mean effect size. The details of individual methods are presented in the Methods section. Their pros and cons are summarized in Supplementary Table S1.

In this paper, we develop a method for the meta-analysis of diverse datasets generated across multiple species. Our method is semi-parametric in nature, requiring fewer assumptions on the distribution of the effect size than a purely parametric approach while retaining better statistical power than a fully non-parametric method. It also 1) defines an effect size that is gene centric, 2) allows for the computation of a mean effect size, and 3) leads to a heterogeneity statistic to test for differences in correlation structures among distinct datasets. Unlike most network alignment algorithms [14],[15],[16],[17],[18] (with the exception of [19]) or connectivity-based approaches [20], our method does not rely on the networks inferred a-priori from individual datasets, but instead focuses on the development of rigorous statistics to test directly the relationship between every gene pair. The simulation results showed that our method is robust against noises. When applied to a human, mouse and rat cross species meta-analysis of liver co-expression networks, we demonstrate that our method out-performs existing methods in identifying functionally coherent gene pairs that are conserved among the three species. Our method also leads to the identification of modules of co-expressed genes that represent core functions of the liver that have been conserved throughout evolution. Both highly replicated and less confident genome-wide association study (GWAS) candidate genes for blood lipid levels are found to be enriched in the conserved modules, providing a systematic way to elucidate the mechanisms affecting blood lipid levels. Application of our test for homogeneity leads to the identification of a single sub-network driven by *ApoE* that distinguishes two nearly identical experimental cross populations whose genetic backgrounds only vary with respect to the gene *ApoE*. We further demonstrate that genes involved in human- or rodent- specific liver interactions tend to be under positive selection throughout evolution. Finally, we identified a human-specific sub-network regulated by *RXRG*, which has been validated to play a different role in hyperlipidemia and Type 2 diabetes between human and mouse. Taken together, our approach represents a novel step forward in integrating gene co-expression networks from multiple large scale datasets to leverage not only conserved information but also differences that are dataset-specific.

## Results

### A semi-parametric method for meta-analysis of gene co-expression networks

The intuition behind our meta-analysis approach in the cross-species setting is that, instead of directly comparing the correlation coefficients of a gene pair as an absolute measure of co-expression, which depends on many features such as sample size, expression dynamics, measurement noise, and confounding factors that are usually not well-controlled among the individual datasets, we measure the co-expression relationship as a *relative* distance with respect to each gene's total relationship to all other genes in each dataset. When the correlation coefficients between a given gene and all other genes were rank-transformed into a uniform distribution, the inter-relationships among the correlations were destroyed. Unlike the previous method [7] we assume the distribution of correlation coefficients of one gene to all other genes follows a normal distribution under the condition that the numbers of samples and genes are large (see Materials and Methods section for details). In fact, for roughly 70–90% of the expression traits in our datasets, the distributions of their correlation coefficients to all other expression traits are well supported as being normal by the Kolmogorov-Smirnov test (Figure S1). Based on this assumption, we define for gene pair (*i,j*) in dataset 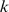, the effect size of its co-expression according to Glass's *d score* definition [21] as:
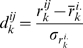
where 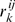 is the correlation coefficient between the expression profiles of (*i,j*) in dataset 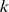, and 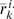 and 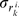 are the mean and standard deviation of the null distribution, respectively, of the correlation coefficients between gene 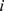 and all other genes. Essentially, by this definition we transform the correlation measure into a relative distance to the gene-centric mean in terms of standard deviation units. This transformation not only normalizes all effect sizes, but also takes into account the context of each gene in individual datasets. It is of further note that our effect size definition is directional, i.e. 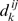 is usually different from 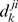 due to differences in the neighborhoods of gene 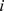 and 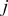. For simplicity, we drop the superscript 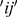 so that 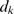 represents the effect size for any gene pair in dataset 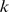.

Using a meta-analysis procedure for *d score* that developed by Hedges and Olkin [8], we can compute the mean effect size as:
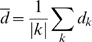
and the standard deviation of the mean effect size as:
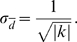
The statistical significance of the mean effect size can then be assessed by forming the Z-score statistic:
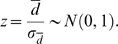



In addition, heterogeneity of the effect sizes across the datasets can be estimated by the statistic
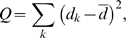
which follows a 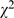 distribution with 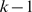 degree of freedom under the null hypothesis of homogeneous effect sizes.

**Figure 1:**
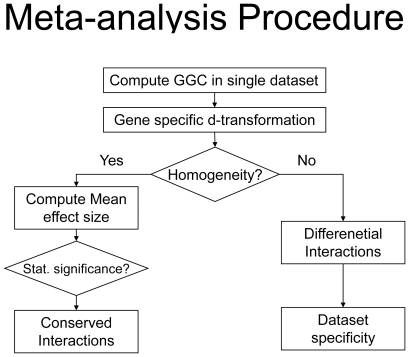
Proposed meta-analysis algorithm flowchart. After calculating all pair-wise gene-gene correlation coefficients (GGC) and transforming them into the proposed d-statistic, the algorithm iterates through every gene-pair one at a time, testing for homogeneity and whether the mean effect size differs significantly from zero.

Given the mean effect size and heterogeneity statistic, a flowchart of our method is summarized in Figure 1. Briefly, the first step begins by computing correlation coefficients for all gene pairs in every dataset. Correlation can be measured by the Pearson or Spearman correlation, depending on the properties of the datasets being analyzed. The method then proceeds by iterating through all gene-pairs one at a time, computing the heterogeneity 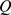 statistic for every gene-pair. If homogeneity is not rejected at a pre-specified significance level, the mean effect size for the gene-pair is computed and tested for deviation from zero. A statistically significant mean effect size is then considered as a conserved co-expression relationship among the datasets being compared. On the other hand, if the homogeneity of the effect sizes is rejected, the gene-pair is considered as a candidate for change in co-expression relationships, termed differential interactions hereafter, between the datasets. In this case, the direction of change can be determined by examining the actual effect sizes in single datasets.

### Simulation studies

**Figure 2:**
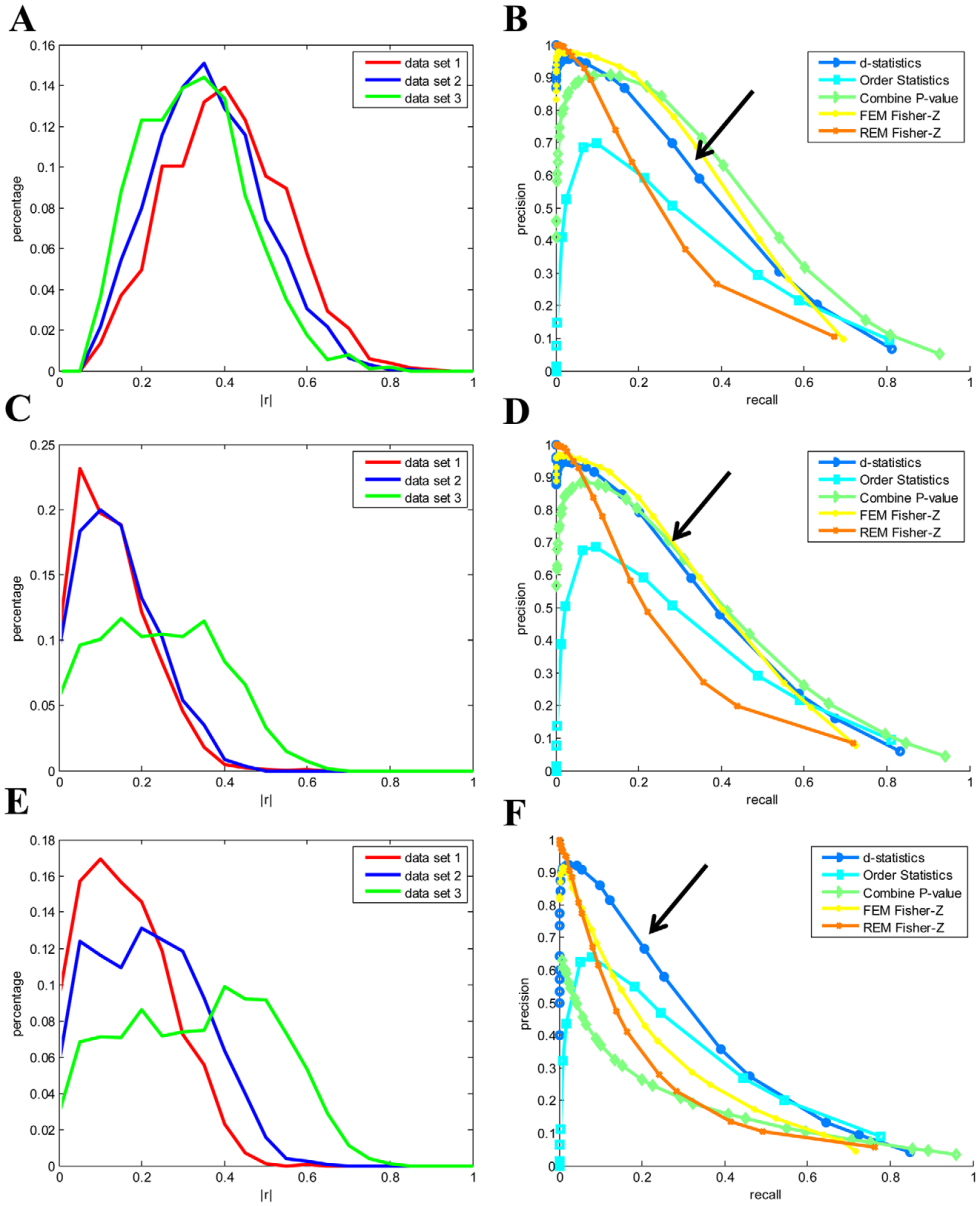
Performance comparison of different meta-analysis methods under different noise conditions. (A) the signal strengths (measured by the correlation between the latent regulators and their downstream genes); (B) the performances of different methods when there was no systematic noise; (C) moderate systematic noises (measured by the correlation between genes and systematic noises); (D) the performances of different methods when the systematic noises were as shown in (C); (E) stronger systematic noises than (C); (F) the performances of different methods when the systematic noises were as shown in (E). FEM Fisher-Z: the fixed effect model based on Fisher-Z transformation; REM Fisher-Z: the random effect model based on Fisher-Z transformation; Combine P-value: combine p-values of Fisher's Inverse 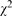 tests; Order Statistic: Order-based non-parametric meta-analysis; d-statistics: the semi-parametric meta-analysis. See Materials and Methods section for details of individual methods.

To compare the performance of our semi-parametric method with the existing parametric and non-parametric methods, we ran several simulations. In each simulation, 3 independent data sets were generated assuming the underlie structure is modular as shown in Figure S2 (see Materials and Methods section for details). There were 150 samples and 2000 genes in each data set. The signal strength is measured by the correlation between the latent regulators and their downstream genes. The signal strengths were different for the 3 simulated data sets, shown in Figure 2A. When there was no systematic noise, the parametric methods (FEM Fisher-Z and combine p-value) performed better than non-parametric method, shown in Figure 2B. It is consistent with other studies' results that there are power losses in general for non-parametric methods [12],[13]. The performance of our semi-parametric method was between the parametric methods and the non-parametric method. It is consistent with the nature that our semi-parametric is a hybrid of parametric and non-parametric methods. It is worth to note that the random effect model (REM Fisher-Z) performed worst among methods tested even though the effect sizes were different as shown in Figure 2A.

When the systematic noises were moderate (measured by the correlation between genes and systematic noises) as shown in Figure 2C, the performances of our semi-parametric method and the parametric methods were similar, shown in Figure 2D. When the systematic noises were stronger (shown in Figure 2E), the performances of parametric methods decreased significantly, and our semi-parametric and non-parametric methods were robust against systematic noises (shown in Figure 2F). Under all conditions, our semi-parametric method performed better than the non-parametric method.

### Conserved interactions among human, mouse and rat

We applied our method to identify conserved co-expression interactions among 6,455 orthologous genes in human, mouse and rat (see Materials and Methods for details about the data, data preparation and orthologous gene identification. The 6,455 genes are listed in Table S2. The 2-D hierarchical clustering views of individual data sets are shown in Figure S3, and ordered sample and gene annotations are listed in Table S3, S4, S5, S6, S7, S8). We used the absolute Spearman correlation coefficient between the expression profiles of a gene pair as the measure of co-expression interaction. By doing this we considered only the magnitude of gene-gene correlation, but not its direction, since the same gene-gene relationship may manifest as either a positively or negatively correlated expression profile due to feedback control [4]. Specifically, our method inferred 20,230 conserved co-expression interactions, covering 4,885 genes, at a p-value cutoff of 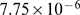, corresponding to a Bonferroni corrected false positive rate of 5% (i.e. 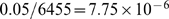) for both effect size and the heterogeneity 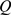. The false discovery rate (FDR) of this result is estimated to be 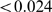 based on a permutation test procedure where sample labels were randomly shuffled for each gene independently in every dataset (see Materials and Methods for details). These conserved interactions represent approximately 2.4–15.2% of the co-expression interactions obtained using single species data, given there were 828,031, 334,721 and 132,884 interactions in human, mouse and rat, respectively, at the same statistical significance *p*-value threshold.

**Figure 3:**
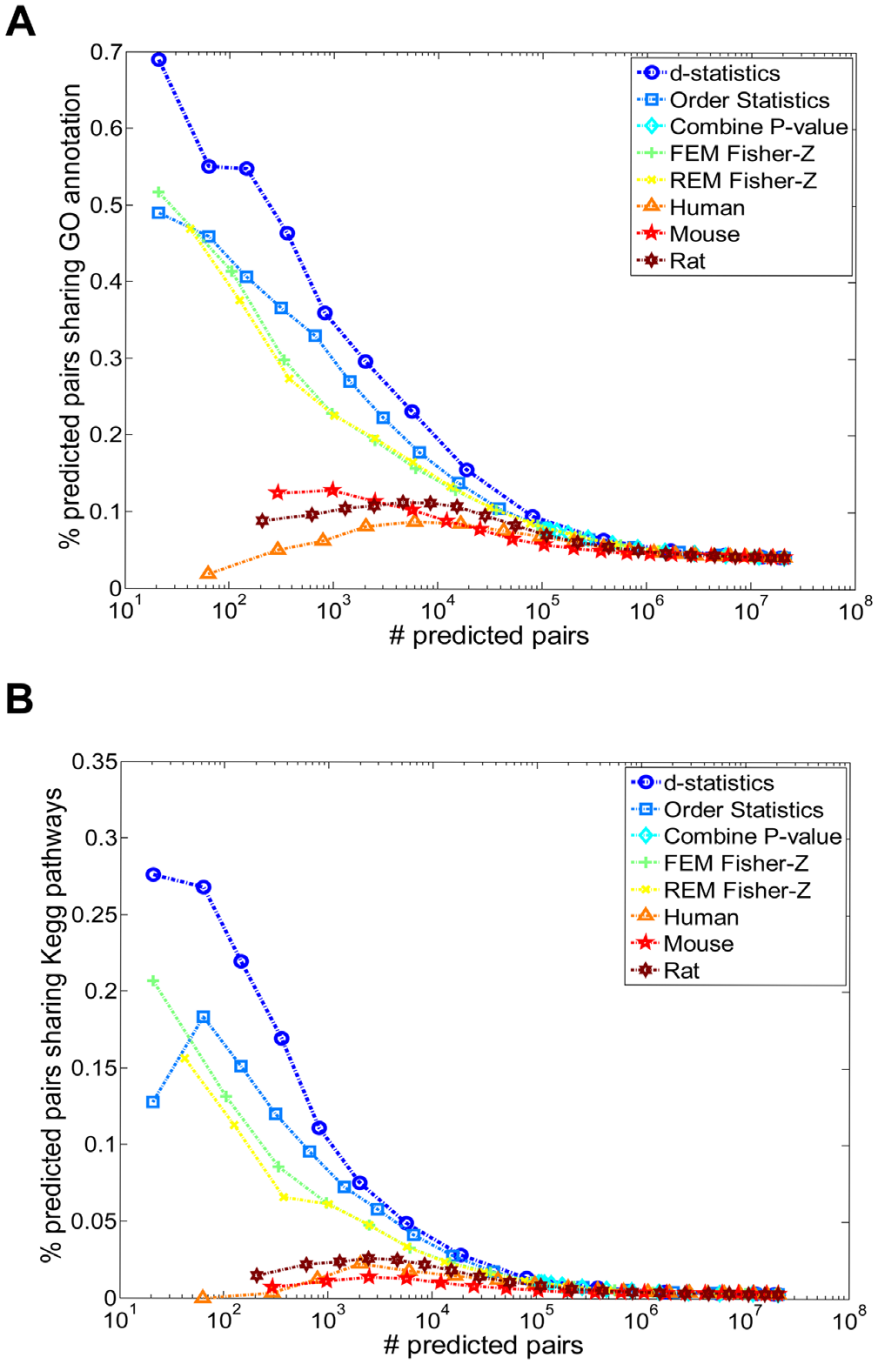
Performance comparison of the proposed method with existing meta-analysis methods, and with co-expression networks reconstructed using single species datasets (i.e. without evolution information), in identifying functionally coherent co-expression interactions. (A) Benchmarking using specific GO biological process categories. (B) Benchmarking using curated KEGG pathways. The abbreviations of different meta-analysis methods are the same as Figure 2.

We benchmarked the performance of our method against existing meta-analysis methods in the literature, as well as against the interactions previously reported for single species co-expression networks [22]. The number of predictions (i.e. conserved interactions) inferred by our method lies in between the numbers predicted by existing parametric and non-parametric meta-analysis methods at a common FDR threshold, shown in Table S9, consistent with the semi-parametric nature of our approach. When only considering the same number of top confident predicted pairs, the qualities of the semi-parametric method were better than other methods in terms of coherences with both Gene Ontology (GO) biological processes and curated KEGG pathways (shown in Table S10). To test the full range of predictions, we generated precision vs. coverage curves for each method by varying the statistical significance thresholds and computing 1) the percent of inferred gene pairs that share a common GO biological process annotation, and 2) the percent of inferred gene pairs that share a common curated KEGG pathway (Figure 3). Two conclusions stand out from these results. First, all meta-analysis methods outperform the inference based only on single species datasets, likely due to the increased precision achieved by incorporating evolutionary information and the added power achieved by integrating multiple datasets. Second, our method clearly outperformed all existing meta-analysis methods across the full spectrum of coverage, but most significantly at the stringent *p*-values. This demonstrates the added value of combining the advantages of existing methods.

We next performed spectral clustering of the orthologous genes based on their interconnectivity in the conserved co-expression network and identified co-expressed gene modules, shown in Figure S4 (see Materials and Methods for the spectral clustering method). Table 1 summarizes the top 13 modules comprised of greater than 20 genes and their enrichment for GO biological process terms. Almost all of the modules are observed to be coherent with respect to some biological processes and many of the indicated processes represent core biological processes in the liver, including immune response (*p*<2.70×10^−43^), carboxylic acid metabolic process (*p*<6.6×10^−16^), and sterol biosynthetic process (*p*<1.9×10^−27^). It is of particular note that these modules differ from modules identified in single species datasets in that the genes in modules of the conserved co-expression network are functionally related based on evolutionary conservation, rather than on correlated gene expression alone.

**Table 1:** Over-represented GO biological process categories for modules identified from the conserved liver co-expression network among human, mouse and rat.

M	GO Process	*P*	*E*	Background Size	Background Overlap	Module Size	Module Overlap
1	Cell-cell signaling	2.60×10^−27^	1.70×10^−23^	5519	417	1024	168
2	Translation	7.10×10^−23^	4.70×10^−19^	5519	182	559	67
3	Ribonucleoprotein biogenesis	2.50×10^−12^	1.60×10^−8^	5519	111	527	37
4	Carboxylic acid metabolic process	4.10×10^−19^	2.70×10^−15^	5519	379	481	88
5	Transcription from RNA polymerase II promoter	5.60×10^−9^	3.70×10^−5^	5519	483	451	76
6	Immune response	2.70×10^−44^	1.70×10^−40^	5519	414	404	119
7	Cell adhesion	2.10×10^−8^	1.40×10^−4^	5519	493	112	30
8	Carboxylic acid metabolic process	1.30×10^−18^	8.40×10^−15^	5519	379	106	38
9	Positive regulation of JNK cascade	3.65×10^−3^	1.00	5519	23	76	3
10	Cell cycle	6.80×10^−23^	4.40×10^−19^	5519	254	74	31
11	Sterol biosynthetic process	3.20×10^−30^	2.10×10^−26^	5519	33	62	19
12	Dlycerophospholipid biosynthetic process	4.00×10^−4^	0.92	5519	34	24	3
13	Regulation of DNA replication	7.64×10^−3^	1.00	5519	41	18	2

Modules are sorted in decreasing order according to their modularity (see Materials and Methods). ‘***P***’ is the nominal FET *p*-value. ‘***E***’ indicates the expected false discovery rate after correcting for multiple testing (i.e. FET *p*-value multiplied by the total number of GO categories tested). Except modules 9, 12 and 13, all permutation adjusted p-values are 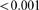.

### Understanding GWAS lipid candidate genes using conserved interactions

Recent human genome-wide association studies have identified many candidate genes affecting blood lipid concentrations. However, the mechanisms by which many of these candidate genes contribute to blood lipid concentration remains unclear [23]. In addition, there are potentially many SNPs with weaker associations to lipid concentration that are difficult or impossible to detect or replicate given the lack of power in current GWAS [24]. Therefore, an open question is whether there are many more genes harboring common variation that affect the polygenetic nature of lipid concentration regulation. Because liver is a key tissue for lipid metabolism, we can use the liver networks to interpret the GWAS results and generate hypothesis regarding the mechanisms of the candidate genes. Toward this end, we selected 30 recently identified lipid-associating loci [25] and assessed the ability of our conserved modules to annotate the 45 candidate causal genes nominated from these 30 loci. Of the 45 candidate genes, 26 have orthologs in human, mouse and rat and were therefore included in our study. Nineteen of these genes reside in human, mouse and rat conserved modules (Table 2), where the putative mechanisms with respect to lipid regulation can be annotated based on the module functions. The results suggest that cellular processes such as sterol biosynthetic process and cell-cell communication are involved in regulating blood lipid concentration. Of particular note is *SORT1*, a gene that resides at the locus most significantly associated with LDL cholesterol [25]. Based on the conserved modules, *SORT1* belongs to module 1, a module enriched for genes involved in cell-cell signaling (*p*-value<6.51×10^−23^). Other candidate genes at lipid associated loci, such as *GALNT2* and *NCAN*, also reside in module 1, suggesting that cell-cell signaling is important for blood lipid regulation. *PCSK9* is clearly annotated as being involved in the sterol biosynthetic process along with *FADS1*, *FADS2*, *HMGCR* and *MVK*. In contrast, only 14 of 26 candidate genes can be annotated based on modules derived from the human co-expression networks alone (Table 2). The annotations of these genes based on the conserved modules are closer to their known functions than ones based on the human modules (shown in Table S11). For example, *MAFB* is annotated as “transcription regulation” based on the conserved modules, but as “carboxylic acid metabolic” based on the human-only modules, whereas its annotation in GO is “positive regulation of transcription from RNA polymerase II promoter”. These examples illustrate how the conserved human, mouse and rat modules can enhance the interpretation of GWAS and the annotation of candidate genes identified from these studies.

**Table 2:** Annotation of GWAS lipid candidate genes using conserved co-expression modules as well as modules based on human data alone.

Conserved Modules				Human Modules			
#	Annot	Size	GWAS Candidate	#	Annot	Size	GWAS Candidate
1	Cell-cell signaling	1211	GALNT2,SORT1,NCAN	1	Carboxylic acid metabolic	1022	ANGPTL3,GALNT2,ABCG8,HMGCR,APOA5,MAFB,HNF4A
2	Translation	665	LIPG	2		802	
3	Ribonucleoprotein biogenesis	619	XKR6	3	Translation	561	MLXIPL,ABCA1,APOE
4	Carboxylic acid metabolic	568	ANGPTL3,GCKR,LDLR	4		275	
5	Transcription regulation	523	TRIB1,ANGPTL4,MAFB	5	Immune response	257	TIMD4
6	Immune response	447	TIMD4	6		189	
7	Cell adhesion	121		7		156	FADS3
8	Carboxylic acid metabolic	110	APOA5,APOE	8		101	
9		95		9		109	
10	Cell cycle	83		10		89	
11	Sterol biosynthetic	67	PCSK9,HMGCR,FADS1,FADS2,MVK	11		81	
12		27		12		66	
13		25		13		81	
				14		82	
				15		48	
				16		44	
				17	Cell proliferation	41	TRIB1,LDLR
				18		44	
				19		51	
				20		49	

**#**: module number; **Annot**: GO annotation categories.

**Figure 4:**
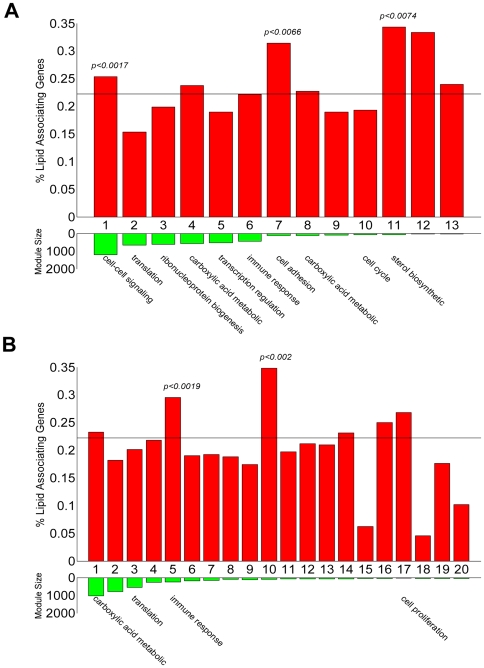
Conserved co-expression modules show better association with human lipid traits. Red bars show the percent of lipid associating genes in each module; the green bars show the module sizes. The dotted horizontal line indicates the background percentage of lipid associating genes. For modules with significantly enriched GO categories, the most representative category is labeled below the green bars. *P*-values above the red bars are the FET *p*-value for modules significantly enriched in lipid-associating genes. (A) Conserved co-expression modules. (B) Modules built from the human gene expression data alone.

Blood lipid concentration regulation is a complex process, involving many different cellular pathways. We have recently demonstrated that common variation of complex traits is caused by networks of genes as opposed to single genes [4]. To assess whether GWAS results associate with entire networks of genes, we overlapped blood lipid concentration results from the Framingham heart study [26] and the Broad Institute lipid study [27] with the human, mouse and rat conserved liver network. In this analysis, we consider a gene as associated with the blood lipid trait if any SNP associated with the trait in these studies lies within 50Kb of the gene. Then, at a *p*-value threshold of 0.001, 22.2% of the genes with human, mouse and rat orthologs are associated with blood lipid concentration in either study. At the same *p*-value cutoff, 19.7% of all human genes in our dataset were associated with blood lipid concentration, suggesting that the lipid concentration regulation mechanism is conserved globally (∼1.13 fold enrichment, Fisher's Exact Test (FET) *p*-value = 5.38×10^−11^, permutation adjusted *p*-value<0.001, Figure S5A). The distribution of genes associated with blood lipid concentration among the modules is shown in Figure 4A. Seven of the 13 modules were observed to have a higher concentration of genes associated with blood lipids than the background. Modules 1, 7 and 11 were significantly enriched for genes associated with blood lipid levels (1.14, 1.41 and 1.55 fold enrichment with FET *p*-values of 1.7×10^−3^, 6.6×10^−3^, and 7.4×10^−3^, respectively). These results suggest that cell-cell signaling, cell-adhesion and sterol biosynthesis pathways are associated with variation in blood lipid concentration regulation in the human population. In contrast, a similar test was applied to modules identified from human expression profile data alone. The module with the highest overlap with genes associated with blood lipid traits was not enriched for a coherent biological process and the module enriched for carboxylic acid metabolism were not significantly enriched for genes associated with blood lipid traits (Figure 4B). We have further showed that these results are not sensitive to the window size around the lipid-associating loci for selecting lipid-associating genes. The trends of the global conservation of lipid-associating genes and results in Figure 4 hold true also for window size of 10K, 20K, 30K and 40K (Table S12 and Figure S6).

Genetic loci associating with blood lipid traits from both Framingham and Broad studies may harbor many genes in each of these regions. Dissecting the true causal genes from those irrelevant ones remains a significant challenge. We have previously shown that cis eSNPs – SNPs that are associated with the mRNA levels of genes residing in the same genomic regions – are enriched for functionally relevant genes associating with the trait of interest [28]. In addition to the cis eSNPs, functionally coherent gene modules, representing the cellular processes associated with the trait of interest, can also help pinpoint the true causal genes. By filtering the Framingham and Broad candidate lipid-associating genes with genes that either 1) harbor a cis eSNP in its vicinity, or 2) belongs to any of the three conserved co-expression modules enriched in lipid-associating genes, the overlap between the two studies becomes more significant than the un-filtered sets, demonstrating the utilities of cis eSNP and conserved co-expression modules in teasing out irrelevant candidate genes (shown in Table 3; in this case, the cis eSNP genes we previously identified from a liver expression study were used [28]). There were 395 genes (Table S13) that are associated with a cis eSNP in the human liver, and are also in the three conserved co-expression modules we identified as associated with the blood lipid trait. These genes represent the most likely causal genes controlling the blood lipid concentration by integrating GWAS candidate loci, human cis eSNP genes and conserved co-expression modules between human and rodent species. Among these genes, four of them, *SORT1*, *FADS1*, *FADS2* and *GALNT2*, are recently reported as candidate genes at highly replicated genetic loci contributing to polygenic dyslipidemia [25]. This result is statistically significant given there are only 26 such candidate genes in our initial set of 6455 orthologous genes between human and rodents (a 2.51-fold enrichment, FET *p*-value<0.0189, permutation adjusted *p*-value<0.015, Figure S5B). These results demonstrate that the combination of multiple types of information can provide an objective way to infer causal genes under the loci of interest.

**Table 3:** Conserved modules and cis eSNPs can help elucidate common candidate genes from GWAS results.

	FHS	Broad	Overlap	*P*
All lipid associating genes	1,142	438	147	2.55×10^−17^
Genes with cis-eSNPs	429	175	58	1.71×10^−27^
Genes in the conserved co-expression modules	297	108	37	1.83×10^−24^

**FHS:** Framingham heart study; **Broad:** Broad Institute lipid study; ***P*:** FET *p*-value for the overlap.

### Differential interactions between F2 mouse cross BXH/wt and BXH/apoe^−/−^

Many factors contribute to the identification of differential interactions between human, mouse and rat, such as evolution differences, genetic background differences, and perturbation differences in the data sets (such as genetic diversity in human liver data vs. diverse compound treatments in rat liver data), to name just a few. As a proof of concept, we applied our meta-analysis approach to identify differential interactions between the liver co-expression networks from two previously reported F2 intercrosses. The first F2 intercross was constructed between C57BL/6J ApoE null (B6.ApoE^−/−^) mice and C3H/HeJ ApoE null (C3H.ApoE^−/−^) mice (referred as BXH/apoe^−/−^) [29]. The second F2 intercross was constructed between C57BL/6J (B6) wild type mice and C3H/HeJ (C3H) wild type mice (referred as BXH/wt) [30]. These two crosses are essentially identical from the standpoint of genetic background, diet, and rearing, except that in one of the crosses the *ApoE* gene is knocked out. Given this single gene difference between the crosses, we hypothesized that differentially connected genes would be enriched for genes associated with *ApoE* related pathways.

Our method identified 500 differentially connected genes involving 1,023 differential interactions between the BXH/wt and BXH/apoe^−/−^ crosses. GO enrichment analysis for this set of genes revealed that the only over represented biological process were those involving *ApoE* [31], albeit these processes are highly overlapping, including the cholesterol metabolic process (4.5% vs. 0.7% background, *p*<5.6×10^−6^), the sterol metabolic process (4.5% vs. 0.9% background, *p*<1.2×10^−4^) and the lipid metabolic process (15.2% vs. 7.2% background, *p*<3.3×10^−4^). Interestingly, no core biological processes in liver that do not involve *ApoE* (e.g., immune response) were enriched, which serves as a negative control for our results. To test whether these differential interactions were mainly driven by expression dynamic changes as the result of the *ApoE* gene knockout, we selected a set of 500 genes with the largest difference in expression variation between the two crosses. GO enrichment analysis revealed no coherent biological functions represented in this set, indicating that the observed network changes could not be explained simply by dynamic differences in gene expression.

**Figure 5:**
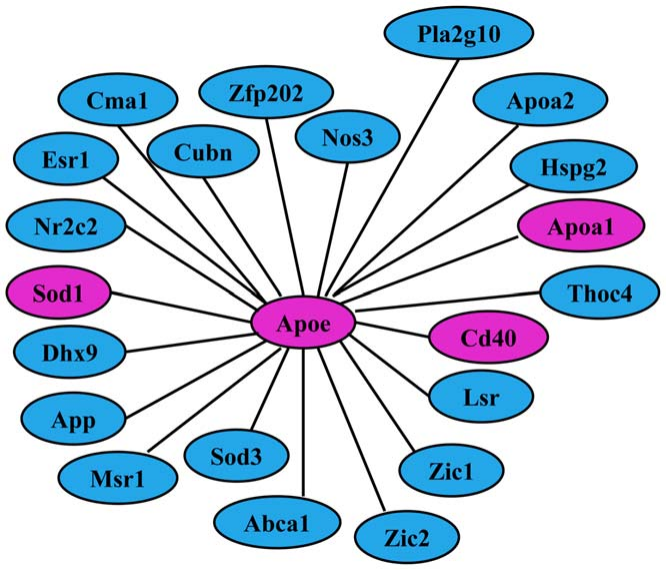
Known protein-protein and protein-DNA interactions around *ApoE* curated from interaction databases and the literature. Nodes colored in pink are those identified by our method as differentially connected between BxH/wt vs. BxH/apoe^−/−^.

We further examined the mouse protein-protein and protein-DNA interaction networks curated from interaction databases and literature, including Ingenuity, GeneGO and HPRD, around the *ApoE* gene. Of the 22 genes in the immediate neighborhood of *ApoE*, including *ApoE* itself, 4 (18.2%) were inferred as differentially connected between the wild type and *ApoE* ^−/−^ crosses, and this proportion was highly significant (∼8.1 fold enrichment, FET *p*-value<1.1×10^−4^, permutation adjusted *p*-value<0.001) (Figure 5 and Figure S5C). Taken together, these results demonstrate the ability of our meta-analysis procedure to dissect differentially regulated pathways around specific molecular perturbations. Although our method is purely expression profile based, it can also recapitulate known physical interactions in the region of the source perturbation, which further supports the validity of our approach.

### Differential interactions between human and rodent species

Differential interactions among diverse organisms can result from true evolutionary differences or from incomplete perturbations in the datasets we examined, leading to reduced expression dynamics in one or both of the interacting genes. Here we assumed that the gene expression system in each species we examined was extensively perturbed, either directly or indirectly (via second or higher order effects). The human samples were collected from more than 400 unrelated individuals, making up an out-bred population comprised of 400 diverse genetic backgrounds. The F2 mice obtained from the BXH crosses represent an in-bred population in which differences in the genetic background of the parental strains are randomly shuffled in each of the individual mice. The rat expression profiles were generated by treating rats with a compendium of drug compounds with various mechanisms of action. Therefore, although liver gene expression in each species is measured under different sets of perturbations, the extensiveness of these diverse perturbations was likely to render that most pathways were perturbed given there are a finite number of pathways.

We carried out the cross-species meta-analysis in a pair-wise fashion to produce human vs. mouse and human vs. rat comparisons. For the human vs. mouse comparison our method identified 8,706 conserved interactions involving 3,205 genes, in addition to 613 differential interactions involving 547 genes. For the human vs. rat comparison, we identified 10,809 conserved interactions among 3,310 genes, as well as 447 differential interactions among 420 genes. All results were obtained using a *p*-value cutoff of 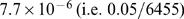.

We further characterized each orthologous gene considered in the comparisons by classifying each gene's involvement in 1) only conserved interactions, 2) at least one differential interaction. Since it has been shown that genes differentially connected in the co-expression and physical interaction networks tend to evolve at different rate [32],[33], we also attempted to characterize the evolutionary rate for each group by measuring the ratio between the rate of non-synonymous to synonymous substitution (Ka/Ks) [34] in the protein coding regions of the respective genes. Interestingly, for both comparisons we found that genes involved in a larger number of differential connections tend to have a higher Ka/Ks ratio (Figure S7). These results suggest that stronger positive selection (or relative weaker negative selection) may lead to new advantages for a given gene by increasing or decreasing the number of its co-expression partners. To further illustrate this point, we expanded our analysis to include genes that are non-orthologous between human and rodents, and tested whether genes that were differentially connected among orthologous genes also tended to have more interactions with non-orthologous genes in a given species, compared to genes involved in only conserved interactions. This was indeed the case when we looked at the ratio of interactions to human-specific genes vs. human-rodent orthologs in the liver co-expression network built from human expression profiles (Figure S8). Taken together, these results demonstrate that positive selection may render a gene the ability to rewire its co-expression connections with evolutionarily conserved partners as well as to add new partners that emerge through speciation.

### Human-specific interactions

One important aspect of understanding the difference in gene expression regulation between human and rodent species is that rodent species (mouse in particular) are frequently used to elucidate the complexity of human diseases. However, there is no guarantee that discoveries made in mouse regarding causes of disease will translate into human systems, so such results can be misleading [35]. In addition to mice being used as a model for human diseases, rats have been established as a critically important model for human drug metabolism and toxicity trials. However, the extent to which toxicity results in rat are faithfully reproduced in humans has not been well characterized [36]. Among the many species-specific variations between human and rodents that may cause such barriers, differential rewiring of the co-expression networks can be an important contributing factor. Understanding species-specific interactions, especially human-specific interactions, is a necessary step to develop relevant animal models for human diseases.

**Figure 6:**
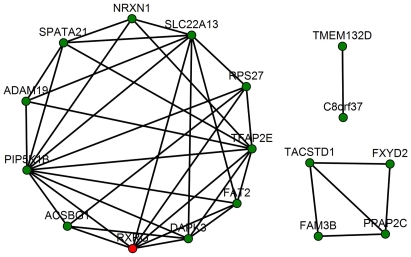
Sub-networks of genes with the most human-specific interactions. Node in red is ***RXRG*.** Nodes are connected by an edge if their co-expression relationship exists only in human, but not in mouse or rat.

Again using the same *p*-value threshold described above, 1,171 differential interactions were identified among the human, mouse and rat liver co-expression networks. An interaction between two genes is considered human-specific if 1) the co-expression relationship between the two genes is significantly different between human and the rodent species based on the heterogeneity test, 2) the correlation *p*-value of the two genes in human is smaller than 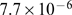, and 3) the correlation *p*-values for the two genes in both mouse and rat are larger than 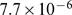. Of the 1,171 differential interactions identified, 163 were human-specific. The top 20 genes with most human-specific interactions are listed in Table S14. These genes are inter-connected to form three sub-networks (Figure 6). The largest sub-network consists of 11 genes, three of which (*PIP5K1B*, *RXRG* and *ACSBG1*) are well known to be involved in lipid metabolism. *RXRG* (retinoid X receptor gamma) emerges as a key regulator of this human-specific sub-network. It is one of the genes with the most predicted human-specific interactions, and 7 out of 8 of its interactions involve other genes also with the most human-specific interactions (*PIP5K1B*, *TFAP2E*, *SLC22A13*, *DAPK3*, *RPS27*, *FAT2* and *ACSBG1*). *RXRG* homozygous mutant mice are normal [37], suggesting that it may not exert any essential function in mouse. However, there are many evidences suggesting that *RXRG* variations in humans are associated with lipid metabolism [38], as well as with glucose and Type 2 diabetes [39]. *RXRG* mutations are the most frequent variations in familial combined hyperlipidemia and are associated with triglycerides and HDL cholesterol [40]. These differences in *RXRG*'s role between human and mouse are consistent with our prediction that there are differences between human and rodents networks around *RXRG*. In addition to *RXRG*'s 8 predicted human-specific interactions with genes having a rodent ortholog, it is also known to be an upstream regulator of *CETP* [41] which has no corresponding ortholog in either mouse or rat. *CETP* encodes a cholesteryl ester transfer protein that plays a key role in regulating HDL cholesterol. Thus it may partially explain *RXRG*'s contribution to lipid metabolism in humans. These results suggest that attention should be paid to retinoid X receptor activities when *CETP* transgenic rodent models are studied.

## Discussion

There are a number of systematic efforts for studying complex human diseases using human samples or animal models. Co-expression networks represent a powerful system-level tool for dissecting the architecture of gene expression, and the complex relationships between genes and disease associated traits. Combining co-expression networks across multiple datasets, especially those measured in common tissues from evolutionarily distant species, has the potential to greatly enhance the power to distinguish true associations among gene expression traits from those spurious interactions picked up by guilt-by-association techniques in single datasets. We presented a novel semi-parametric meta-analysis method to combine multiple high dimensional datasets from different species. When applied to the human, mouse, and rat liver co-expression networks, our method out-performed all existing methods with respect to the degree of biological coherence reflected by the identified gene pairs. Using the co-expression network conserved across human, mouse and rat, we identified cell-cell signaling, cell-adhesion and sterol biosynthesis processes as the primary mechanisms represented by GWAS gene candidates associated with blood lipid levels.

In comparing human and rodent co-expression networks we found that ∼10% of the gene-gene co-expression relationships were conserved, in accordance with a recently published comparative analysis of human and mouse gene expression patterns [42]. The conserved interactions could be organized into gene modules that corresponded to core pathways that are critical to normal cellular functions, and therefore are likely to lead to disease if disrupted. Knowledge of the conserved interactions between human and rodent species has the potential to facilitate studies of human disease using rodent models. When we combined conserved liver modules with cis-eSNP information and GWAS results, we identified a list of 395 candidate genes regulating blood lipid levels. Six of these genes (*MTHFR*, *PEX5L*, *CPE*, *LIPA*, *UCP3* and *PLIN*) have previously been shown to have mutant phenotypes in mouse that involve abnormal lipid levels. Systematic testing of the genes in this set using experimental techniques such as siRNA in cell-based systems could provide further confirmation of their involvement in regulating blood lipid concentrations.

Under a unified framework, our method also allows the identification of gene-gene relationships that differ significantly between datasets. The sensitivity of our method to identify dataset-specific biological perturbations was well highlighted by the identification of a single sub-network driven by *ApoE* that was able to distinguish two nearly identical experimental cross populations whose genetic backgrounds were identical with the exception of *ApoE* (knocked out in one of the crosses). This type of network comparisons can help characterize network plasticity due to evolution. We have shown that genes involved in such differential interactions between human and rodents are likely to be under positive selection for gaining or losing co-expression partners. Given that only ∼10% of gene-gene relationships are conserved between these diverse species, divergence in gene expression are likely to be more extensive than genome sequences. It has shown through a chip-chip study that the overlap of transcription factor binding sites is only about 20% across 3 different yeast species where sequence differences are about 0.05% [43]. In some cases, the promoter regions are identical across genomes of 3 yeast species, transcription factors only bound in one species but not others. Thus, variation in transcription regulation is much larger than sequence variation. There could be other factors affecting conversation of pairwise relationship in different data sets, such as 1) inadequate expression dynamics in those parts of the system that lack targeted perturbations, and 2) experimental and technological noise that subdue the real changes in co-expression.

In addition to the meta-analysis methods we compared, there are graphic model-based meta-analysis or Bayesian meta-analysis methods which have been applied to gene expression data in several studies [44],[45]. The performance of Bayesian meta-analysis depends on priors tuning. If noninformative priors are used, then the Bayesian meta-analysis is close to the random effect model. Even through effect sizes are clearly different in our simulated data and empirical data, the mixed effect model performed worse than the fixed effect model. On the other hand, our meta-analysis method is robust across multiple conditions without any tuning of parameters. In addition, the Bayesian meta-analysis is away more computation intensive than the method we proposed so that we did not include it in our comparison.

Meta analysis of co-expression networks we proposed here allow us to compare co-expression networks constructed from data sets of heterogeneous experimental settings. If experimental settings are similar, then direct comparison of signature sets can also provide insights of conserved mechanisms at system levels. For example, a set of periodically expressed genes in *H. sapiens*, *S. ceravisiae*, *S. pombe* and *A. thaliana* was defined and then orthologs of these genes were compared to see whether they peaked during the same phase of cell cycle [46]. However, in our datasets, experimental conditions were different - the variances of human and mouse liver expression data were due to naturally occurred genetic variation, whereas those in the rat liver expression data were due to diverse compound treatment. Therefore, there is no common way to define gene signatures across different data sets that can be compared directly.

Gene expression is one type of high throughput data that can be leveraged to systematically study human diseases. There are many other types of high-dimensional data to which our method could be applied, including protein-protein interaction, protein expression, metabolite expression, and Chip-on-chip data. Further developments are needed to combine these different types of data across different species. Nevertheless, even at its current stage, our method has been successful in identifying mechanisms that are common between and distinct to human and rodent species, which provides the potential to aid in the drug development process.

## Materials and Methods

### Datasets

We profiled 423 human liver samples [28], 382 liver samples of rats treated with different classes of drugs [47], 300 mouse liver samples from an F2 murine intercross between C57BL/6J ApoE null (B6.ApoE^−/−^) and C3H/HeJ ApoE null (C3H.Apo E^−/−^) (referred as BXH/apoe^−/−^) [29], and 321 mouse liver samples from an F2 intercross between C57BL/6J (B6) wild type mice and C3H/HeJ (C3H) wild type mice (referred as BXH/wt) [30]. For every gene in each expression dataset, the expression values were mean-subtracted and then divided by the standard deviation. Missing values were imputed by the robust regression based the expression of the gene most correlated to the query gene expression.

### Orthologous gene identification between human, mouse and rat

Orthologous gene pairs between human, mouse and rat represented on microarrays were identified by taking the reciprocal best hit using BLASTN with an E-value cutoff of 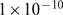. This resulted in 8,767 orthologous pairs identified between human and mouse, 6,934 between human and rat, and 10,185 between mouse and rat. There were 6,455 orthologous genes common to all three species, which were selected for subsequent analysis (Table S2).

### Meta-analysis methods

#### Combine P-value (combine p-values of Fisher's Inverse 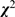 tests)

This method computes a combined statistic 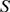 from the p-values of the correlation coefficients obtained from 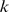 individual datasets as, 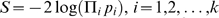. Under general conditions the statistic 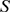 follows a 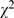 distribution with 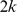 degrees of freedom under the joint null hypothesis of no correlation.

#### FEM Fisher-Z (Fixed effect models based on Fisher's Z-statistic)

In this method proposed by Hedges and Olkin [8] and Rosenthal and Rubin [9], correlation coefficient 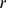 in dataset is first to the Fisher's z-statistic by
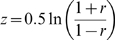
It follows then 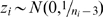 where 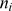 is the sample size of dataset 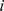. The mean effect size can be computed as:
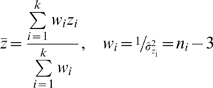
The mean effect size can be converted back to correlation coefficient using the reverse Fisher's z formula:
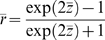
And its statistical significance can be assessed with effective sample size 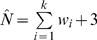.

In addition, the homogeneity of the effect sizes across dataset can be evaluated using the following 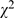 statistic:
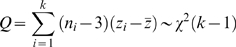



#### REM Fisher-Z (Random effect model based on Fisher's z-statistic)

The random effect model differs from the fixed effect model above in how weight of individual 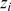 is calculated:
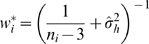


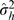
 is heterogeneity variance and can be estimated by:
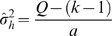
where
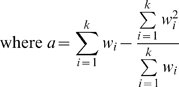
and 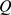 is the heterogeneity statistic introduced above. The remaining procedures of the method are the same as those in the fixed effect model.

#### Order Statistics (Order-based non-parametric meta-analysis)

In this method [7], Pearson correlation coefficients of expression profiles between every gene pair were computed in each dataset and then transformed into rank ratios (i.e. rank divided by the total number of gene pairs) according to their correlations with all other genes. A probabilistic test based on order statistics was then applied to evaluate the probability of observing a particular configuration of ranks across the different datasets by chance. Briefly, let 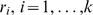, be the rank ratio for a gene pair in dataset 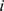, which follows a 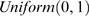 distribution. The joint distribution of the rank ratios across the 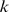 datasets is then:
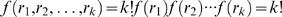
It can be further shown that the joint cumulative distribution is:
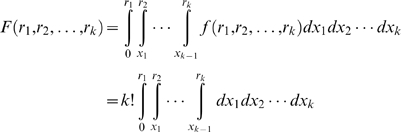
And the p-value associated with a particular rank configuration can then be computed as 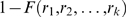.

### Distributions of correlation coefficients

To estimate the significance of a correlation coefficient 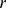, we generally convert 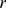 to 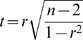 which follows a student t-distribution with 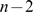. When the sample size 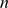 is large enough, 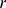 is approximately normally distributed [11]. However, convergence of the distribution is very slow and it is said to be unwise to assume its normality for *n*<500 [48].

The assumption for estimating the Pearson correlation coefficient distribution is that all vector pairs are independently and identically distributed. However, this may not hold true in practice such as microarray experiments due to the facts that 1) probes for two genes on the same chip may be correlated because that are subjected to many common noises and biases, and 2) two unrelated genes in a biological network are still remotely connected so that they can not be completely independent. As a result, not all gene pairs are independent, thus their expected correlation coefficient is not necessary zero. In this case, an empirical null distribution is needed. We note that empirical null distributions are different for each gene/probe so that there are 6455 null distributions instead of one global null distribution. We assume the empirical null distribution of all pair-wise correlation coefficients as a normal distribution based on the central limit theorem, which states that the mean of sufficiently large number of independent random variables will be approximately normally distributed [49].

In summary, we assume the empirical null distribution of pair-wise correlation coefficients as a normal distribution under two conditions: (1) the sample size is large so that the variation of 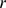 is small; (2) the number of genes under study is large so that the central limit theorem can be applied.

We note that our sample sizes are in the range of 300–500, which are out of the recommended range for normal assumption. However, our normal assumption for correlation distributions of our data is supported by the Kolmogorov-Smirnov test of normality. The sample sizes of the data sets we simulated are 150. We checked the distributions of correlation coefficients of each individual gene, and found that correlation coefficients for over 98% of genes are normally distributed. For the empirical data sets, correlation coefficients for over 70% of genes are normally distributed, which is shown in Figure S1.

### Gene expression data simulation

We assume the underlie system consists of 2000 genes which are divided into10 functional modules and 1 null module, as shown in Figure S2. Each functional module consists of 100 genes and the null module consists of 1000. For simplicity, we assume each gene in a functional module is linearly related to a latent regulator and is simulated as 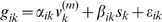, where 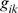 is a 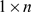 vector (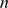, the sample size) representing the expression of gene 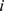 (which belongs to the functional module 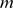) in data set 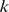. 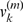 is a 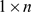 vector for the latent variable 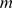 in data set 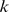. 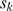 is a 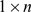 vector representing systematic noise in the data set 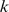. 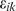 is the random noise. Genes in the null model are not related and are simulated as 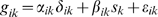, where 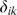 is a random 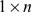 vector. 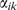 and 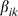 are regression coefficients representing the strengths of the signal and the systematic noise, respectively. The latent variables 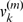, the random signals 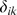, systematic noise 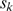 and random noise 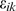 are all assumed to be normally distributed with mean 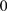 and different variances. The coefficients 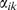 in are constrained by the strength of correlation 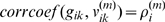. The sign of the coefficient 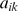 was randomly assigned. It is similar for 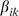. We assume 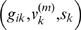 are jointly normally distributed, we can write their covariance as 
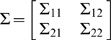
, where 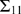 is of size 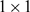, 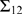 is of size 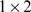, 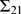 is of size 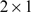, and 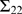 is of size 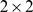. The regression coefficients, 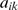 and 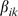 are then given by 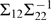, and the error term, 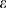, is normally distributed with mean 0 and variance 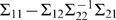.

### Comparing the reconstructed coexpression networks with the true network: recall and precision

To assess the goodness of the reconstructed coexpression networks derived from different meta-analysis methods, they were compared to the true network, which was formed by linking all genes in the same functional module as defined in the simulation process. We define the “goodness” of the reconstructed network in terms of its accuracy, which is measured by two parameters. The first parameter is defined as the precision of the network: 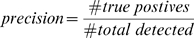, which is the proportion of detected interactions that actually exist in the true network. Precision corresponds to specificity and is equal to one minus the false positive rate (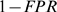). The second parameter is defined as the recall of the network: 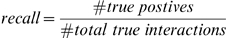, which is the proportion of total interactions in the true network that are detected in the reconstructed network. Recall corresponds to sensitivity and is equal to one minus the false negative rate (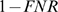), which is also known as the true positive rate (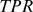). The recall and precision for a perfectly reconstructed network are equal to 1.

The central figure of merit used to evaluate and compare the coexpression networks derived from different meta-analysis methods (with respect to the true network) is the recall vs. precision curve, which can be considered as a variation of the traditional Receiver Operator Characteristic (ROC) curve. ROC curves are generated by plotting the true positive rate (TPR) against the false positive rate (FPR). The area under the ROC curve (AUC) is then a measure of how the constructed network compares to the true network. The larger the AUC, the better the constructed network compares to the true network, where the maximum AUC is 1, indicating that the constructed network perfectly matches the true network. Qualitatively the recall vs. precision curve is equivalent to the ROC curve in that if the AUC for one network is greater than (or less than) the AUC of a second network with respect to one of plot types, that same relationship will hold for the other plot type. We opted to use the recall vs. precision plots over the ROC plots as the figure of merit because recall and precision are the more standard measures used in the network reconstruction community.

### Estimating FDR for the meta coexpression network

The false discovery rate (FDR) of our meta-analysis results was estimated using permutation test procedures. For the conserved interactions, null datasets were created by randomly and independently shuffling the expression values of all genes in each dataset, thus breaking the inter-gene relationships while keeping intact the expression mean and standard deviation of the genes in every dataset. For the differential interaction, we generated the null datasets by shuffling the dataset membership of the samples, so that the permuted datasets are essentially random subsets of the total original samples. The same meta-analysis procedure was applied to both the original datasets as well as the permuted ones. The FDR was then computed as the ratio between the number of inferences made from the permuted datasets (i.e. false discoveries) over the number of inferences made from the original datasets (i.e. total predictions).

### GO coherence and enrichment analysis

Only GO biological process categories with fewer than 1,500 genes (according to human annotations) were included for analysis, precluding non-specific categories, such as metabolic process, from entering the analysis. All GO enrichment analyses were performed using the Fisher's exact test, with all 6,455 orthologous genes forming the background gene set for the human, mouse and rat comparisons. For the BXH/wt vs. BXH/apoe^−/−^ analysis, the background set was comprised of all genes represented on the microarray used in the study.

### Spectral clustering and module identification

To partition the network of genes obtained from our procedures into modules of genes, we employed the divide-and-merge methodology of clustering [27], where a top-down divide phase based on a theoretical spectral algorithm [50] was used to obtain a clustering tree, and a bottom-up merge phase was used to parse the clustering tree to obtain a partition of the genes (gene modules) that optimized a certain objective function. We used the modularity function [51] to identify modules in the human-mouse-rat conserved network. The definition of modularity from the cited references is provided here for completeness. Let 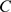 be a partition of the genes in a network into clusters 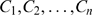. Then,
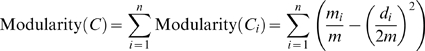
where 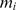 is the number of edges between two genes that both belong to 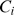, 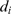 is the sum of the number of neighbors of all genes in 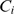, and m is the number of edges in the whole network.

## Supporting Information

Figure S1 Normality check of the distributions of all pair-wise Pearson correlation coefficients by Kolmogorov-Smirnov (KS) test in (A) human, (B) mouse and (C) rat data. The red dotted lines represent the statistical significance cutoff for rejecting the normality assumption.
(0.02 MB PDF)

Figure S2 The modular structure used in gene expression simulation. The network consists of 10 functional modules and 1 null module. Genes in each functional module are regulated by a latent regulator.
(0.02 MB PDF)

Figure S3 2-D Hierarchical clustering results of 3 liver data sets. 6455 orthologous genes are on the horizontal axis, experiments are on the vertical axis. (A) for the human liver data; (B) for the mouse liver data; (C) for the rat liver data. The ordered sample annotations in the vertical axis and the ordered gene symbols in the horizontal axis for each figure are listed in Tablea S9, S10, S11, S12, S13, S14.
(0.26 MB PDF)

Figure S4 Visualization of modules identified by spectral clustering on the connectivity matrix of conserved interactions among human, mouse and rat. Orthologous genes among the three species are on both rows and columns. A back dot represents a conserved interaction between the corresponding gene pairs. Colored squares along the diagonal indicate identified modules, which are numbered in decreasing order according their modularity (see main text Materials and Methods).
(0.02 MB PDF)

Figure S5 Permutation test results for enrichment analyses. (A) Null distribution (blue bars) of the number of lipid associating genes by randomly select 1000 sets of 6455 genes, and the statistic calculated based on the 6455 orthologous genes (red line). (B) Null distribution (blue bars) of the number of validated lipid associating genes by randomly select 1000 sets of 395 genes, and the statistic calculated based on the 395 cis-eSNP genes belonging to the conserved modules (red line). (C) Null distribution (blue bars) of the number of ApoE first neighbor genes among 1000 randomly selected sets of 500 genes, and the statistic calculated based on the 500 differentially connected genes (red line).
(0.02 MB PDF)

Figure S6 Module enrichment of lipid-associating genes at different windows sizes. The interpretation of the figures is the same as that of main text Figure 4.
(0.03 MB PDF)

Figure S7 Conserved and differential interactions between human and rodent species. (A) Human vs. mouse comparison. (B) Human vs. rat comparison. Numbers in the parenthesis are number of genes in each category. P-values were computed using Kruskal Wallis non-parametric test of equal medians (median Ka/Ks for the “Conserved Only” and “greater or euqal to 1 Different” categories are respectively 0.090 and 0.103 for the human vs. mouse comparison; and 0.086 and 0.101 for the human vs. rat comparison).
(0.02 MB PDF)

Figure S8 In comparison with genes involved in only conserved interactions between human and rodents (box on the left), genes having human-specific interactions with human-rodent orthologs (box on the right) display a higher ratio of interactions to human-specific genes vs. human-rodent orthologs in the human liver co-expression network. Top 161 genes from each group were used for plotting the two boxes. Human liver co-expression network was built as previously reported. For genes in each group, its ratio between the numbers of interactions to human-specific vs. human-rodent orthologs is plotted on the Y-axis.
(0.02 MB PDF)

Table S1 Summary of the pros and cons of existing meta-analysis methods
(0.03 MB PDF)

Table S2 6455 orthologous genes among human, mouse and rat used in the analysis.
(1.42 MB XLS)

Table S3 Ordered annotations of human liver samples shown in Figure S3A.
(0.01 MB TXT)

Table S4 Ordered human gene symbols for genes shown in Figure S3A.
(0.05 MB TXT)

Table S5 Ordered annotations of F2 mouse liver samples shown in Figure S3B.
(0.00 MB TXT)

Table S6 Ordered mouse gene symboles for genes shown in Figure S3B.
(0.05 MB TXT)

Table S7 Ordered annotations of rat liver samples shown in Figure S3C.
(0.03 MB TXT)

Table S8 Ordered rat gene symbols for genes shown in Figure S3C.
(0.05 MB TXT)

Table S9 Number of predicted gene pairs that are significantly co-regulated at FDR <0.024 for all existing meta-analysis methods and the proposed method. ‘%GO’ indicates the percent of gene pairs sharing a common specific Gene Oncology biological process category. The background percentage is 0.0413 for randomly selected gene pairs that share common GO biological processes. FEM Fisher-Z: the fixed effect model based on Fisher-Z transformation; REM Fisher-Z: the random effect model based on Fisher-Z transformation; Combine P-value: combine p-values of Fisher's Inverse χ^2^ tests; Order Statistic: Order-based non-parametric meta-analysis; d-statistics: the semi-parametric meta-analysis. See Methods section for details of individual methods.
(0.01 MB PDF)

Table S10 The qualities of top predicted pairs based on existing meta-analysis methods and the proposed method. 20,230 was chosen based on the proposed semi-parametric method at the false positive rate 0.05. ‘%GO’ indicates the percent of gene pairs sharing a common specific Gene Oncology biological process category. ‘%KEGG’ indicates the percent of gene pairs sharing a common KEGG pathway. The abbreviations of different meta-analysis methods are the same as Table S9.
(0.01 MB PDF)

Table S11 Comparison of annotations for GWAS candidate genes based on the conserved modules or human modules. The annotation based on conserved module agrees better with the annotation based on the gene's Gene Ontology annotation.
(0.01 MB PDF)

Table S12 Enrichment of lipid-associating genes among orthologous genes between human and rodents. Lipid-associating genes are selected at different window size around a lipid-associating loci in Framingham and Broad studies.
(0.03 MB PDF)

Table S13 List of 395 candidate lipid-associating genes by integrating GWA results, cis eSNP and conserved co-expression modules. RefSeq IDs, symbols and chromosomal location for each gene are shown in the table.
(0.06 MB XLS)

Table S14 Top 20 genes with the most human-specific co-expression interactions. The numbers of interactions among themselves are also shown.
(0.02 MB PDF)
