# Supplementary figures and images for: Meta-analysis of Inter-species Liver Co-expression Networks Elucidates Traits Associated with Common Human Diseases
Source: PLoS Comput Biol. 2009 Dec 18;5(12):e1000616. doi: 10.1371/journal.pcbi.1000616 (PMC2787626; doi:10.1371/journal.pcbi.1000616)

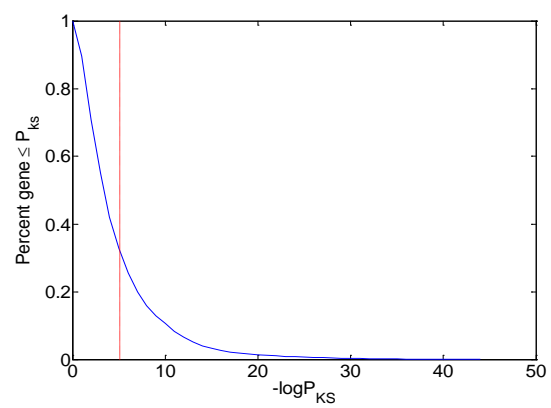

(A)

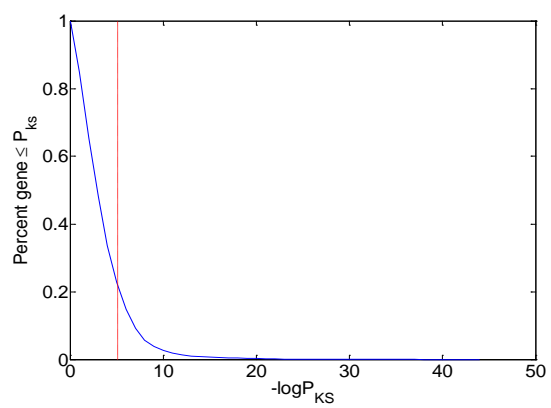

(B)

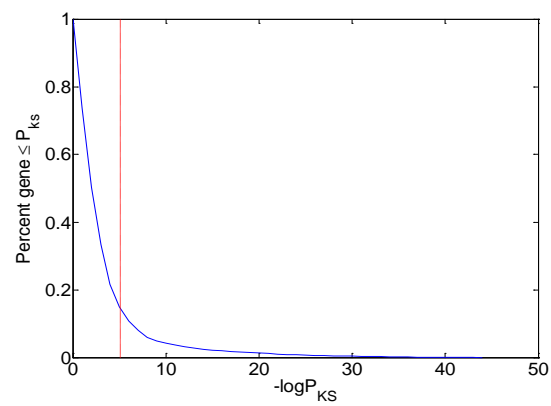

(C)

Supplement: Figure S1 — Normality check of the distributions of all pair-wise Pearson correlation coefficients by Kolmogorov-Smirnov (KS) test in (A) human, (B) mouse and (C) rat data. The red dotted lines represent the statistical significance cutoff for rejecting the normality assumption. (0.02 MB PDF) [file pcbi.1000616.s001.pdf]

## Expression data simulation

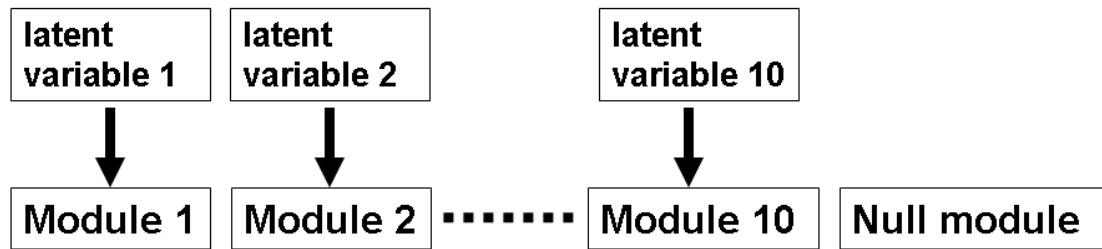

Supplement: Figure S2 — The modular structure used in gene expression simulation. The network consists of 10 functional modules and 1 null module. Genes in each functional module are regulated by a latent regulator. (0.02 MB PDF) [file pcbi.1000616.s002.pdf]

(A)

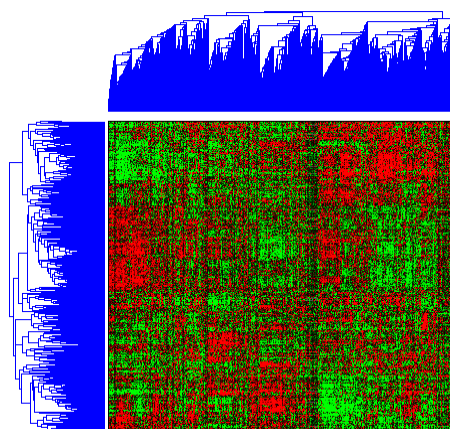

(B)

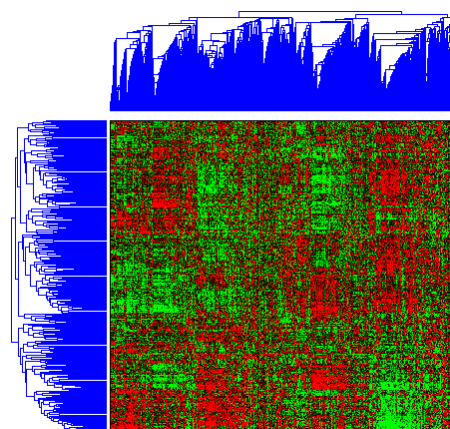

(C)

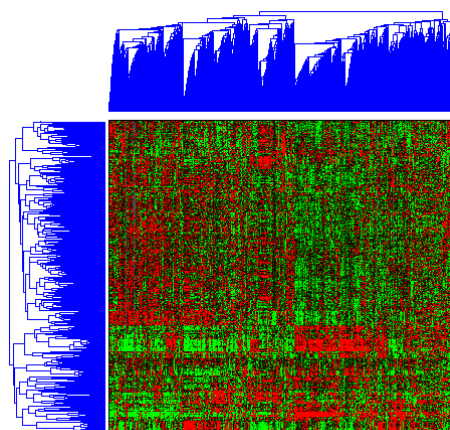

Supplement: Figure S3 — 2-D Hierarchical clustering results of 3 liver data sets. 6455 orthologous genes are on the horizontal axis, experiments are on the vertical axis. (A) for the human liver data; (B) for the mouse liver data; (C) for the rat liver data. The ordered sample annotations in the vertical axis and the ordered gene symbols in the horizontal axis for each figure are listed in Tablea S9, S10, S11, S12, S13, S14. (0.26 MB PDF) [file pcbi.1000616.s003.pdf]

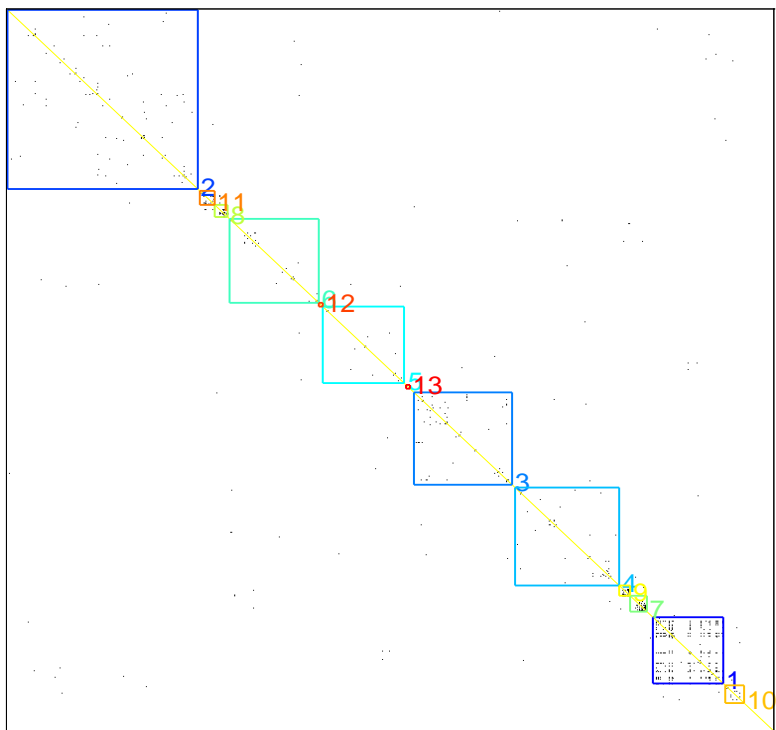

Supplement: Figure S4 — Visualization of modules identified by spectral clustering on the connectivity matrix of conserved interactions among human, mouse and rat. Orthologous genes among the three species are on both rows and columns. A back dot represents a conserved interaction between the corresponding gene pairs. Colored squares along the diagonal indicate identified modules, which are numbered in decreasing order according their modularity (see main text Materials and Methods). (0.02 MB PDF) [file pcbi.1000616.s004.pdf]

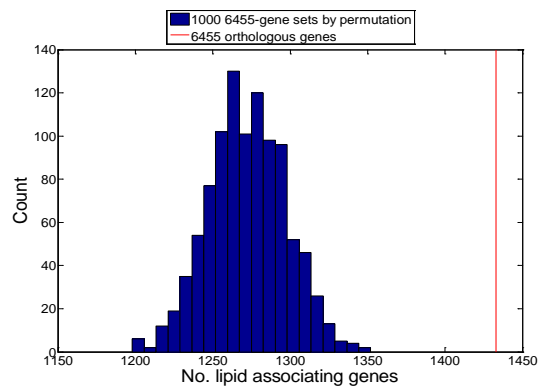

(A)

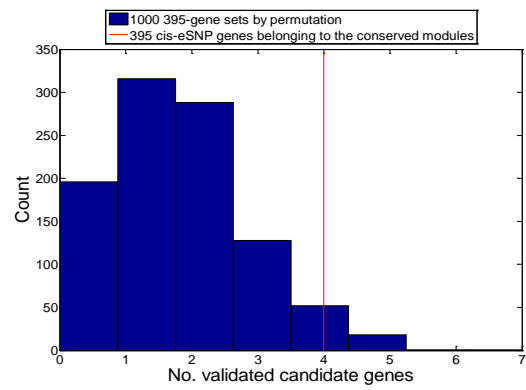

(B)

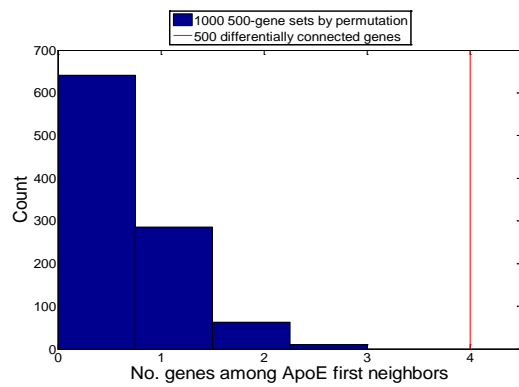

(C)

Supplement: Figure S5 — Permutation test results for enrichment analyses. (A) Null distribution (blue bars) of the number of lipid associating genes by randomly select 1000 sets of 6455 genes, and the statistic calculated based on the 6455 orthologous genes (red line). (B) Null distribution (blue bars) of the number of validated lipid associating genes by randomly select 1000 sets of 395 genes, and the statistic calculated based on the 395 cis-eSNP genes belonging to the conserved modules (red line). (C) Null distribution (blue bars) of the number of ApoE first neighbor genes among 1000 randomly selected sets of 500 genes, and the statistic calculated based on the 500 differentially connected genes (red line). (0.02 MB PDF) [file pcbi.1000616.s005.pdf]

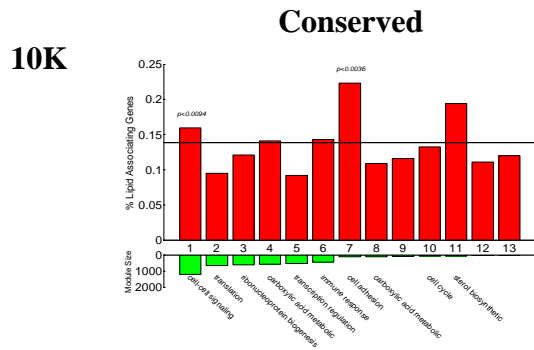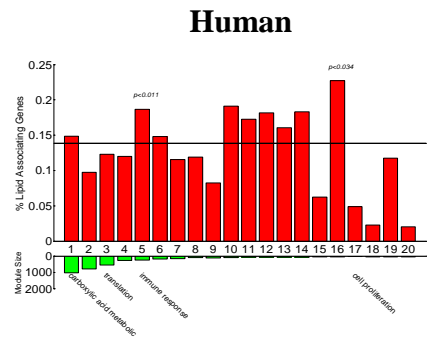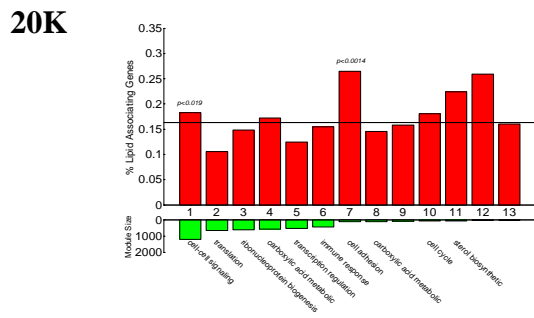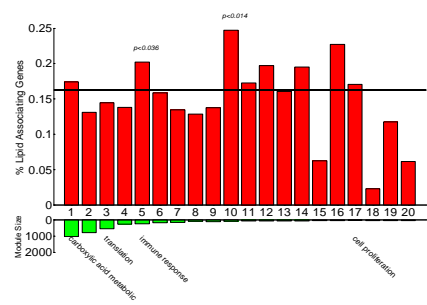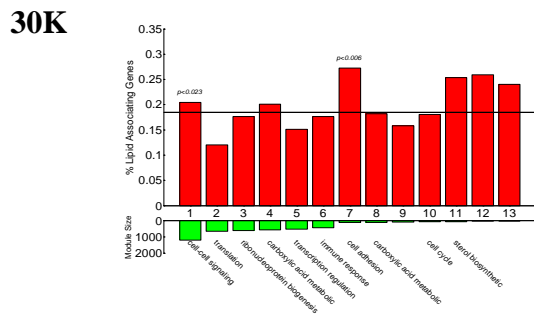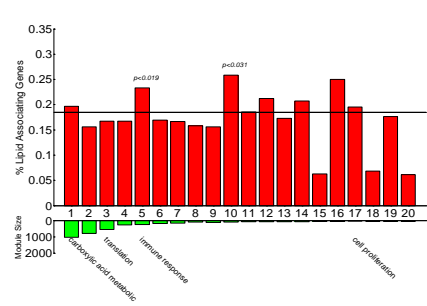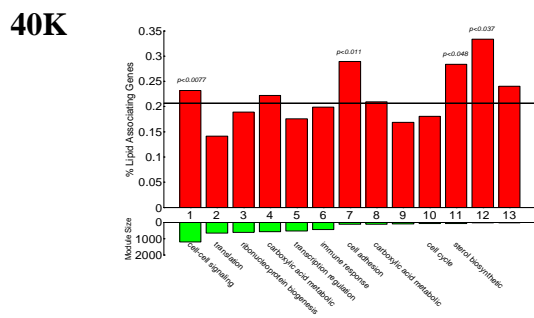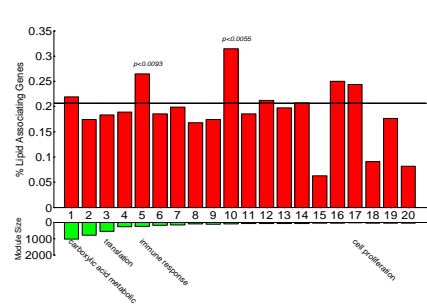

Supplement: Figure S6 — Module enrichment of lipid-associating genes at different windows sizes. The interpretation of the figures is the same as that of main text Figure 4. (0.03 MB PDF) [file pcbi.1000616.s006.pdf]

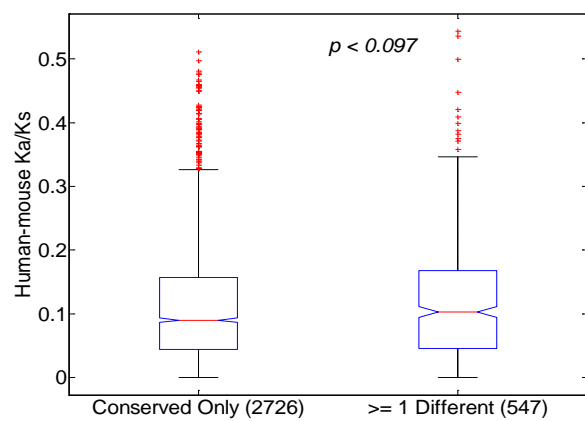

**A**

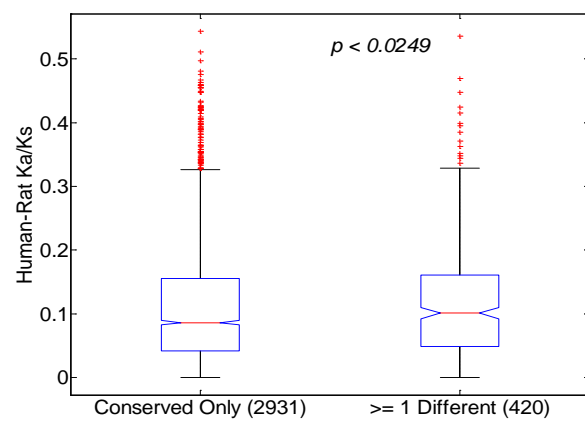

**B**

Supplement: Figure S7 — Conserved and differential interactions between human and rodent species. (A) Human vs. mouse comparison. (B) Human vs. rat comparison. Numbers in the parenthesis are number of genes in each category. P-values were computed using Kruskal Wallis non-parametric test of equal medians (median Ka/Ks for the “Conserved Only” and “greater or euqal to 1 Different” categories are respectively 0.090 and 0.103 for the human vs. mouse comparison; and 0.086 and 0.101 for the human vs. rat comparison). (0.02 MB PDF) [file pcbi.1000616.s007.pdf]

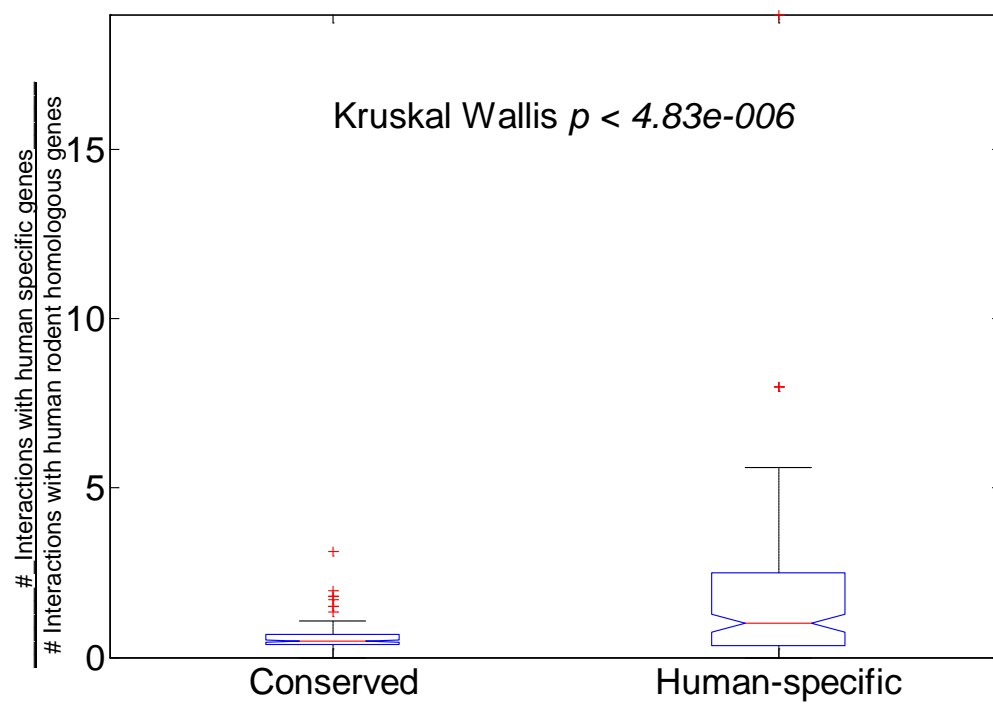

Supplement: Figure S8 — In comparison with genes involved in only conserved interactions between human and rodents (box on the left), genes having human-specific interactions with human-rodent orthologs (box on the right) display a higher ratio of interactions to human-specific genes vs. human-rodent orthologs in the human liver co-expression network. Top 161 genes from each group were used for plotting the two boxes. Human liver co-expression network was built as previously reported. For genes in each group, its ratio between the numbers of interactions to human-specific vs. human-rodent orthologs is plotted on the Y-axis. (0.02 MB PDF) [file pcbi.1000616.s008.pdf]
