# Supplementary material for: Meta-analysis of Inter-species Liver Co-expression Networks Elucidates Traits Associated with Common Human Diseases
Source: PLoS Comput Biol. 2009 Dec 18;5(12):e1000616. doi: 10.1371/journal.pcbi.1000616 (PMC2787626; doi:10.1371/journal.pcbi.1000616)
Supplement: Table S1 — Summary of the pros and cons of existing meta-analysis methods (0.03 MB PDF) [file pcbi.1000616.s009.pdf]

Method	Pros	Cons
Combining P-values [1]		• Over non-conservative
Fisher-Z [2]	• Increased power<br>• Mean effect size<br>• Heterogeneity estimate	• Globally defined effect size<br>• Assume correlations are comparable across different systems<br>• REM has less assumptions but with dramatically reduced power
Order statistics [3]	• Assumption free<br>• Effect size is gene context specific	• Less power<br>• Lack of the concept of a mean effect size<br>• No heterogeneity measurement

References:

1. Hong F, Breitling R (2008) A comparison of meta-analysis methods for detecting differentially expressed genes in microarray experiments. Bioinformatics 24: 374-382.

2. Hu P, Greenwood CM, Beyene J (2005) Integrative analysis of multiple gene expression profiles with quality-adjusted effect size models. BMC Bioinformatics 6: 128.

3. Stuart JM, Segal E, Koller D, Kim SK (2003) A Gene-Coexpression Network for Global Discovery of Conserved Genetic Modules. Science 302: 249-255.
