# Supplementary material for: Meta-analysis of Inter-species Liver Co-expression Networks Elucidates Traits Associated with Common Human Diseases
Source: PLoS Comput Biol. 2009 Dec 18;5(12):e1000616. doi: 10.1371/journal.pcbi.1000616 (PMC2787626; doi:10.1371/journal.pcbi.1000616)
Supplement: Table S9 — Number of predicted gene pairs that are significantly co-regulated at FDR <0.024 for all existing meta-analysis methods and the proposed method. ‘%GO’ indicates the percent of gene pairs sharing a common specific Gene Oncology biological process category. The background percentage is 0.0413 for randomly selected gene pairs that share common GO biological processes. FEM Fisher-Z: the fixed effect model based on Fisher-Z transformation; REM Fisher-Z: the random effect model based on Fisher-Z transformation; Combine P-value: combine p-values of Fisher's Inverse χ2 tests; Order Statistic: Order-based non-parametric meta-analysis; d-statistics: the semi-parametric meta-analysis. See Methods section for details of individual methods. (0.01 MB PDF) [file pcbi.1000616.s017.pdf]

| Meta Method | # Predictions | %GO |
|---|---|---|
| Order statistics | 16,188 | 0.1433 |
| Combine p-value | 3,163,556 | 0.0458 |
| FEM Fisher-Z | 2,093,089 | 0.0458 |
| REM Fisher-Z | 670,051 | 0.0435 |
| d-statistics | 20,230 | 0.1568 |
