# Supplementary material for: Meta-analysis of Inter-species Liver Co-expression Networks Elucidates Traits Associated with Common Human Diseases
Source: PLoS Comput Biol. 2009 Dec 18;5(12):e1000616. doi: 10.1371/journal.pcbi.1000616 (PMC2787626; doi:10.1371/journal.pcbi.1000616)
Supplement: Table S10 — The qualities of top predicted pairs based on existing meta-analysis methods and the proposed method. 20,230 was chosen based on the proposed semi-parametric method at the false positive rate 0.05. ‘%GO’ indicates the percent of gene pairs sharing a common specific Gene Oncology biological process category. ‘%KEGG’ indicates the percent of gene pairs sharing a common KEGG pathway. The abbreviations of different meta-analysis methods are the same as Table S9. (0.01 MB PDF) [file pcbi.1000616.s018.pdf]

**Meta Method**	**# Interactions**	**%GO**	**%KEGG**
d-statistics	20230	15.7	2.8
Order Statistics	20230	13.4	2.6
Combine P-value	20230	7.5	1.2
FEM Fisher-Z	20230	10	1.4
REM Fisher-Z	20230	7.4	0.8
