# Supplementary material for: Meta-analysis of Inter-species Liver Co-expression Networks Elucidates Traits Associated with Common Human Diseases
Source: PLoS Comput Biol. 2009 Dec 18;5(12):e1000616. doi: 10.1371/journal.pcbi.1000616 (PMC2787626; doi:10.1371/journal.pcbi.1000616)
Supplement: Table S11 — Comparison of annotations for GWAS candidate genes based on the conserved modules or human modules. The annotation based on conserved module agrees better with the annotation based on the gene's Gene Ontology annotation. (0.01 MB PDF) [file pcbi.1000616.s019.pdf]

GWAS Gene	Annotation based on conserved module	Annotation based on human module	GO Biological Process (if the conserved and human module provides different annotation)
ANGPTL3	carboxylic acid metabolic	carboxylic acid metabolic	
APOA5	carboxylic acid metabolic	carboxylic acid metabolic	
APOE	carboxylic acid metabolic	translation	lipid metabolic
GALNT2	cell-cell signaling	carboxylic acid metabolic	protein amino acid O-linked glycosylation via serine/threonine
HMGCR	sterol biosynthetic	carboxylic acid metabolic	cholesterol biosynthetic process
LDLR	carboxylic acid metabolic	cell proliferation	cholesterol metabolic process
MAFB	transcription regulation	carboxylic acid metabolic	positive regulation of transcription from RNA polymerase II promoter
TIMD4	immune response	immune response	
TRIB1	transcription regulation	cell proliferation	negative regulation of smooth muscle cell proliferation; negative regulation of transcription factor activity
