# Supplementary material for: Meta-analysis of Inter-species Liver Co-expression Networks Elucidates Traits Associated with Common Human Diseases
Source: PLoS Comput Biol. 2009 Dec 18;5(12):e1000616. doi: 10.1371/journal.pcbi.1000616 (PMC2787626; doi:10.1371/journal.pcbi.1000616)
Supplement: Table S12 — Enrichment of lipid-associating genes among orthologous genes between human and rodents. Lipid-associating genes are selected at different window size around a lipid-associating loci in Framingham and Broad studies. (0.03 MB PDF) [file pcbi.1000616.s020.pdf]

Window Size	Lipid associating genes among orthologs	# ortholgos	Lipid associating genes in human	# human genes	% among orthologs	% among all human genes	FET p-value
10K	894	6455	1718	16464	0.138	0.104	2.00E-30
20K	1051	6455	2203	16464	0.163	0.134	1.30E-18
30K	1194	6455	2583	16464	0.185	0.157	1.10E-15
40K	1335	6455	2971	16464	0.207	0.180	9.30E-13
50K	1433	6455	3244	16464	0.222	0.197	5.40E-11
