# Supplementary material for: Meta-analysis of Inter-species Liver Co-expression Networks Elucidates Traits Associated with Common Human Diseases
Source: PLoS Comput Biol. 2009 Dec 18;5(12):e1000616. doi: 10.1371/journal.pcbi.1000616 (PMC2787626; doi:10.1371/journal.pcbi.1000616)
Supplement: Table S14 — Top 20 genes with the most human-specific co-expression interactions. The numbers of interactions among themselves are also shown. (0.02 MB PDF) [file pcbi.1000616.s022.pdf]

Accession ID	Gene Symbol	No. human-specific interactions	No. Interactions among top 20
NP_003549	PIP5K1B	18	11
NP_848643	TFAP2E	13	9
NP_004247	SLC22A13	10	9
NP_001009598	RXRG	8	7
NP_859072	FOXR1	8	0
NP_001339	DAPK3	8	7
NP_940948	SPATA21	5	5
NP_001021	RPS27	5	5
NP_004792	NRXN1	5	4
NP_001438	FAT2	5	5
NP_001671	FXYD2	5	1
NP_597705	TMEM132D	5	1
NP_478066	FAM3B	5	1
NP_060484	BCL11A	4	0
NP_002345	TACSTD1	4	2
NP_075525	ADAM19	4	4
NP_003595	IRS4	4	0
NP_808880	C8orf37	4	1
NP_055977	ACSBG1	4	4
NP_872299	---	4	4
